# Neuroendocrine and sympathetic responses to an orexin receptor antagonist, SB-649868, and Alprazolam following insulin-induced hypoglycemia in humans

**DOI:** 10.1007/s00213-014-3520-7

**Published:** 2014-04-26

**Authors:** Ameera X. Patel, Sam R. Miller, Pradeep J. Nathan, Ponmani Kanakaraj, Antonella Napolitano, Philip Lawrence, Annelize Koch, Edward T. Bullmore

**Affiliations:** 1 Brain Mapping Unit, Behavioral and Clinical Neuroscience Institute, University of Cambridge, Cambridge, CB2 3EB UK; 2 Clinical Unit Cambridge, GlaxoSmithKline, Addenbrooke’s Centre for Clinical Investigation, Cambridge, UK; 3 School of Psychology and Psychiatry, Monash University, Melbourne, Australia; 4 Quantitative Sciences India, GlaxoSmithKline Pharmaceuticals Ltd, Bangalore, India

**Keywords:** SB-649868, Alprazolam, Orexin, Antagonist, Insulin, Hypoglycemia, Randomized controlled trial

## Abstract

**Rationale:**

The orexin-hypocretin system is important for translating peripheral metabolic signals and central neuronal inputs to a diverse range of behaviors, from feeding, motivation and arousal, to sleep and wakefulness. Orexin signaling is thus an exciting potential therapeutic target for disorders of sleep, feeding, addiction, and stress.

**Objectives/methods:**

Here, we investigated the low dose pharmacology of orexin receptor antagonist, SB-649868, on neuroendocrine, sympathetic nervous system, and behavioral responses to insulin-induced hypoglycemic stress, in 24 healthy male subjects (aged 18–45 years; BMI 19.0–25.9 kg/m^2^), using a randomized, double-blind, placebo-controlled, within-subject crossover design. Alprazolam, a licensed benzodiazepine anxiolytic, was used as a positive comparator, as it has previously been validated using the insulin tolerance test (ITT) model in humans.

**Results:**

Of the primary endpoints, ITT induced defined increases in pulse rate, plasma cortisol, and adrenocorticotropic hormone in the placebo condition, but these responses were not significantly impacted by alprazolam or SB-649868 pre-treatment. Of the secondary endpoints, ITT induced a defined increase in plasma concentrations of adrenaline, noradrenaline, growth hormone (GH), and prolactin in the placebo condition. Alprazolam pre-treatment significantly reduced the GH response to ITT (*p* < 0.003), the peak electromyography (*p* < 0.0001) and galvanic skin response (GSR, *p* = 0.04) to acoustic startle, the resting GSR (*p* = 0.01), and increased appetite following ITT (*p* < 0.0005). SB-649868 pre-treatment produced no significant results.

**Conclusion:**

We concluded that the ITT model may be informative for assessing the effects of drugs directly acting on the neuroendocrine or sympathetic nervous systems, but could not be validated for studying low dose orexin antagonist activity.

**Electronic supplementary material:**

The online version of this article (doi:10.1007/s00213-014-3520-7) contains supplementary material, which is available to authorized users.

## Introduction

The orexin-hypocretin system is fundamentally important in regulating a diverse range of behaviors from feeding, motivation and arousal (Sakurai 2007), to sleep and wakefulness (Hagan et al. 1999; Sutcliffe and de Lecea 2000). In addition, lateral hypothalamic orexinergic neurons have been shown to respond to peripheral metabolic signals including blood pH, ghrelin, leptin, and glucose, indicating that these neurons could be crucial in coupling energy homeostasis to vigilance states (Yamanaka et al. 2003).

Since the early studies of narcolepsy in animals (Lin et al. 1999; Chemelli et al. 1999), a number of reports have implicated the orexin system in endogenously regulating the stability of arousal, and its dysregulation in anxiety and panic-like behaviors (Johnson et al. 2010; Li et al. 2010). Several lines of evidence suggest that orexinergic neurons are central components of the stress response via activation of the hypothalamus-pituitary-adrenal (HPA) axis. These include histological (Blanco et al. 2003; Lopez et al. 1999), in vitro (Nanmoku et al. 2002; Sakamoto et al. 2004; Samson et al. 2002; Kuru et al. 2000), and behavioral stress paradigm data (Martins et al. 2004; Reyes et al. 2003), with a number of additional studies suggesting a direct role for the corticotrophin-releasing factor (CRF) peptidergic system in mediating the effects of orexin on the HPA axis (Samson et al. 2002; Ida et al. 2000a, 2000b; Jászberényi et al. 2000; Winsky-Sommerer et al. 2004). Furthermore, there is evidence that both orexin receptor 1 (OxR1) (Johnson et al. 2010) and orexin receptor 2 (OxR2) antagonists (Chang et al. 2007) can inhibit stress-induced adrenocorticotropic hormone (ACTH) responses, and panic-like behaviors in rats (respectively), indicating a role for both receptors in dysregulated stress responses.

Clinical development of orexin receptor antagonists in phase I and II has focused on insomnia, using relatively high doses to induce sleep. SB-649868, an orally active non-selective OxR1 and OxR2 antagonist (Hagan et al. 1999; Faedo et al. 2012), has hypnotic efficacy from 20 mg per day orally, as characterized by increased sleep time and reduced latency to persistent sleep. However, concerns over potential toxicity from prolonged use of high doses in animal studies, and inter-individual variability in pharmacokinetic parameters, have limited the dose of SB-649868 that can be used in humans. Given this, and the potential therapeutic value of the orexin target, it was thus considered important to evaluate whether this orexin antagonist had pharmacodynamic effects in humans at doses lower than those previously studied.

One of the most powerful methods for studying low dose antagonist pharmacology is under conditions of high endogenous agonist release. Orexin signaling can be stimulated experimentally in a number of ways including psychological, physical, and pharmacological/endocrine stress paradigms; hypercapnic challenge; and hypoglycemia. Ethical stress paradigms in animals can produce robust activation of the HPA axis (Winsky-Sommerer et al. 2004; Chang et al. 2007), but only modest activation in humans (Gaab 2002). Furthermore, such paradigms are prone to poor test-retest reliability. Similarly, while pre-clinical studies have shown that orexinergic neurons are sensitive to CO_2_ levels and pH (Williams et al. 2007), both well known chemical triggers of anxiety, use of CO_2_ inhalation methods to enhance arousal and anxiety in humans have produced modest and variable results (Bailey et al. 2007; Gorman et al. 1997; Poma et al. 2005), and HPA axis activation has been inconsistently reported (van Duinen et al. 2004). In contrast, hypoglycemia has been shown to robustly increase orexinergic neuron activation (Sakurai et al. 1998; Moriguchi et al. 1999; Cai et al. 2001) and orexin levels (Moriguchi et al. 1999; Cai et al. 1999, 2001; Griffond et al. 1999), specifically, orexin A (Liu et al. 2001). These effects are thought to be mediated by “glucose-sensing” neurons (Anand et al. 1964; Oomura et al. 1969; Ouedraogo et al. 2003). In the brain, these are thought to be lateral hypothalamic orexinergic neurons, after a number of studies showed that their firing is glucose-inhibited, and conversely activated under conditions of systemic hypoglycemia (Sakurai et al. 1998; Moriguchi et al. 1999; Cai et al. 2001). More recently, it has been shown that physiological fluctuations in blood glucose concentrations can directly modulate the firing of orexinergic neurons (Burdakov et al. 2005), suggesting that normal variations in the body’s energy resources could be translated into appropriate behavioral states, including arousal (Adamantidis et al. 2007).

A well-established method for inducing hypoglycemia and HPA axis activation is the insulin tolerance test (ITT). Insulin-induced hypoglycemia has been shown to increase ACTH, cortisol, growth hormone (GH), and catecholamine release in a number of studies (Greenwood et al. 1966; Plotsky et al. 1985; Fish et al. 1986; Giordano et al. 2003). The increase in ACTH is thought to be mediated by CRF, as immunization against CRF abolishes the ACTH response to ITT in rats (Plotsky et al. 1985; Caraty et al. 1990). CRF is in turn thought to mediate the effects of orexin on the HPA axis (Samson et al. 2002; Ida et al. 2000a, 2000b; Jászberényi et al. 2000), perhaps by direct innervation of orexinergic neurons (Winsky-Sommerer et al. 2004). In addition, ITT has been shown to increase c-fos expression in lateral hypothalamic orexinergic neurons (Cai et al. 2001), which is coupled to an increase in activity of these neurons, suggesting that insulin-induced hypoglycemia is able to robustly activate the orexin system.

The aim of the present study was to investigate the low dose effects of the OxR1 and OxR2 antagonist, SB-649868, on the HPA axis, specifically, its effects on the sympathetic nervous system (SNS) and neuroendocrine responses to hypoglycemia. The idea was to investigate whether SB-649868 could have effects on these systems by inhibiting abnormally activated orexin signaling, such as is thought to occur in anxiety or panic disorders (Li et al. 2010; Johnson et al., 2012a, 2012b). Alprazolam, a licensed benzodiazepine anxiolytic (and GABA_A_ receptor modulator), was used as a positive comparator. It has been previously validated using the ITT model in a placebo-controlled experimental medicine study, and was shown to attenuate increases in ACTH, GH, and adrenaline (Giordano et al. 2003). To this effect, we studied the effects of pre-treatment with SB-649868, alprazolam, and placebo on a range of neuroendocrine and SNS markers in response to ITT in young, healthy volunteers.

## Materials and methods

### Study design

This study (GSK #115268) used a randomized, double-blind, placebo-controlled, within-subject crossover design. The study was conducted in two cohorts with an interim analysis separating cohorts 1 and 2. Each subject attended up to three testing sessions, each separated by a minimum 2-week washout period. At each session, subjects received either of the following: SB-649868 10 mg, alprazolam 0.02 mg/kg (to the nearest 250 μg; Giordano et al. 2003), or a placebo, orally, in accordance with the drug regime they had been randomly assigned. Drug conditions were crossed over for future sessions. Alprazolam 0.02 mg/kg was used here as a positive control, in order to validate the insulin-induced hypoglycemia model, as it has been previously used to study the effects of hypoglycemia on neuroendocrine and sympathetic nervous system (SNS) responses in humans, at this dose (Giordano et al. 2003).

The study was specifically designed to test the effects of SB-649868 at sub-hypnotic doses. This was motivated partly by concerns about potential toxicity and pharmacokinetic variability of this molecule at high doses, and partly by the intention to investigate the possibility that SB-649868 might have benefits for patients with abnormally activated orexin signaling associated with increased sympathetic tone and/or hypercortisolemia, such as is thought to occur in anxiety or panic disorders (Johnson et al. 2010, 2012a; Li et al. 2010). It was decided on the basis of prior data that doses greater than 10 mg were likely to be associated with hypnotic effects (Bettica et al. 2012a, b) and therefore 10 mg was chosen as the starting dose. The study was designed so that the dose could be adaptively reduced in the light of interim analysis, after collection of data from cohort 1 (*N* = 12). However, when no significant treatment effects were demonstrated by the interim analysis, we opted, per protocol, to increase the sample size to *N* = 24, still testing for effects at a dose of 10 mg, to mitigate the risk of type II error. When no significant treatment effects were demonstrated by analysis of the full sample, we opted, per protocol, to stop the study.

**Fig. 1:**
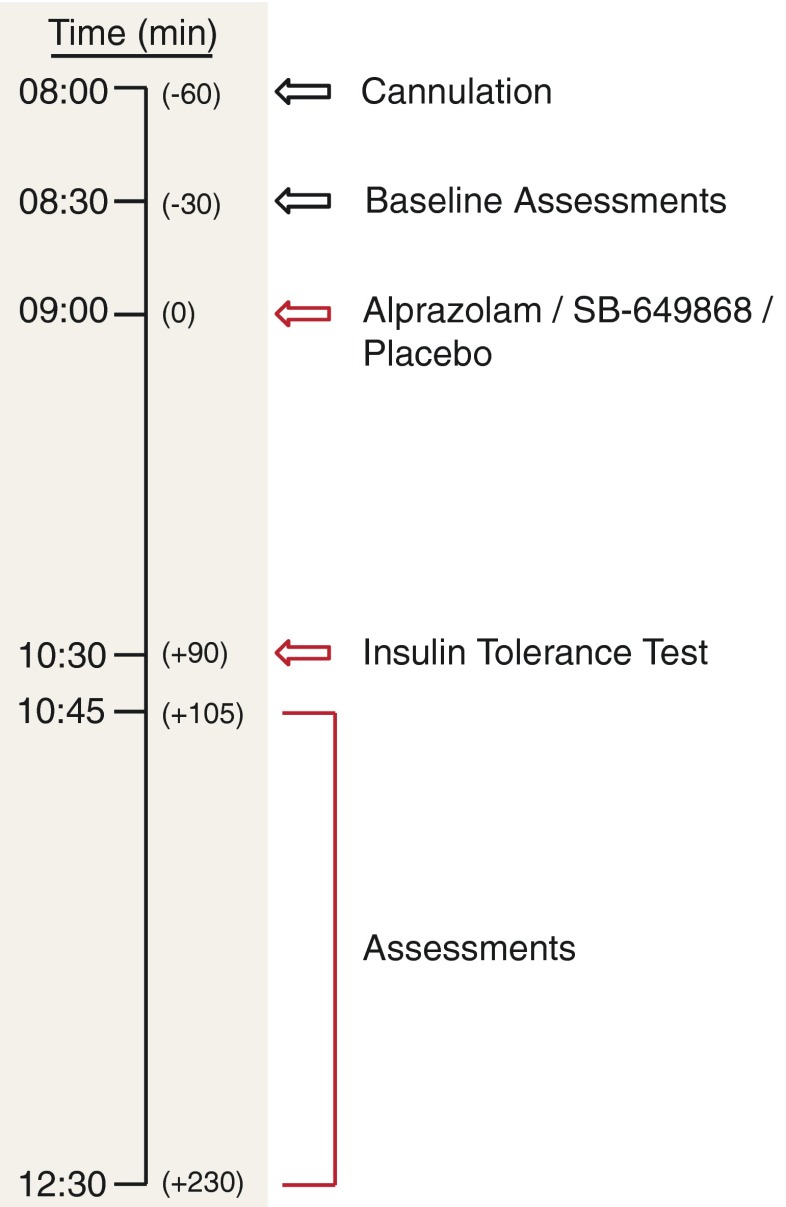
Overview of study testing protocol. Conditions were crossed over, such that subjects attended up to three testing sessions, with a minimum 2-week washout period, each time receiving either placebo, SB-649868 (10 mg) or alprazolam (0.02 mg/kg)

Testing sessions started between 0845 and 0915 h following an overnight fast of 9 hours. Thirty to 60 min prior to dosing, subjects were cannulated and began a schedule of continuous cardiac monitoring, and venous blood sampling for glucose, study endpoints, and pharmacokinetic assays. Subjects were dosed at time *t* = 0 min with either SB-649868, alprazolam, or placebo. At *t* = 90 min, an intravenous bolus of 0.1 U/kg insulin (ITT) was administered (Fish et al. 1986; Giordano et al. 2003). Blood glucose concentrations were monitored every 5 min from 10 to 45 min after insulin injection (*t* = 100 to 135 min), then every 15 min until *t* = 270 min (3 hours after the start of the ITT). At *t* = 210 min, the ITT was concluded and participants were offered an *ad libitum* choice of food and drink. Cardiac monitoring continued until normalization of blood glucose concentrations, and less frequent venous blood sampling until 1600 h. A summary of the testing procedure can be found in Fig. 1.

The protocol was reviewed and approved by the Welwyn Ethics Committee, and the study was conducted in accordance with ICH Good Clinical Practice guidelines, and the guiding principles of the 2008 Declaration of Helsinki. All subjects provided written informed consent prior to testing.

### Endpoints

Primary endpoints were divided into markers of HPA axis activation (plasma ACTH (pmol/L) and cortisol (nmol/L) concentrations), and markers of SNS activation (pulse rate (beats/min) and mean arterial pressure (MAP, mmHg)). Secondary neuroendocrine markers included plasma glucose (mmol/L), adrenaline (nmol/L), noradrenaline (NA, nmol/L), growth hormone (GH, μg/L), prolactin (μg/L), and luteinizing hormone (LH, IU/L) concentrations. Corresponding secondary SNS markers included resting galvanic skin response (GSR, μS) over 4 min, respiratory rate (breaths/min), and acoustic startle response using GSR (μS) and surface electromyography (EMG, μV). The acoustic startle comprised 10 pseudo-randomized bursts of white noise, at 100–110 dB, lasting 50 ms each, over the course of 15 min.

### Subjects

Twenty-four healthy male subjects (aged 18–45 years inclusive; BMI 19.0–25.9 kg/m^2^) were initially recruited in two separate cohorts of 12 subjects each. The final cohort comprised 33 subjects, including 9 replacements (see Section 3.1). Eligible participants underwent pre-study screening 30 days prior to the first dose to rule out any previous, or current, medical or psychiatric condition which may have compromised subject or investigator safety, and/or the interpretation of results. Additionally for this study, the following endocrine criteria were set: fasting plasma insulin <60 pmol/L, fasting plasma glucoses <7.0 mmol/L, and morning plasma cortisol >100 nmol/L.

### Analysis

All endpoints in figures are shown as adjusted group means (adjusted for group size and baseline differences) of absolute values representing maximum change from pre-ITT baseline (∆max), or as areas under the curve (AUC) calculated by integration of values from the pre-ITT baseline (*t* = 90 min) until the end of data collection for that endpoint. Statistical analysis was conducted using standard parametric ANOVA or ANCOVA. Data are plotted ± 95 % confidence intervals (95 % CI). Statistical comparison of side effects between groups was conducted using Fisher’s exact test.

## Results

### Subject response to ITT

Of the 12 subjects enrolled in cohort 1, 2 subjects were withdrawn after failing to achieve a post-ITT reduction in blood glucose level to ≤ 2.2 mmol/L (the concentration required for adequate HPA axis and SNS activation; Fish et al. 1986; Giordano et al. 2003). One subject was replaced for all three sessions, and 1 subject was replaced for sessions 2 and 3. Of the 12 subjects enrolled in cohort 2, 1 subject was withdrawn pre treatment, 4 subjects were withdrawn after failing to achieve a post-ITT reduction in blood glucose level to ≤ 2.2 mmol/L, and 1 subject was withdrawn for an adverse event deemed unrelated to the study drug. Of these, 2 subjects were replaced for all 3 sessions, 2 subjects were replaced for sessions 2 and 3, and 1 subject for session 3 alone. Where subjects failed to achieve an adequate reduction in blood glucose concentration following the ITT, glucose concentrations were within 0.6 mmol/L of the target, and therefore these data were used in the final dataset. Subjects were replaced following an inadequate post-ITT reduction in blood glucose, on the premise that they were more likely to not achieve the desired reduction in blood glucose concentration in subsequent sessions. The analysis dataset thus comprised 80 sessions (26 placebo, 30 SB-649868, and 24 alprazolam sessions) from 33 subjects.

### Side effects

Subjects experienced typical hypoglycemic symptoms in response to the ITT, such as sweating, increased appetite, and increased heart rate. In addition, a number of drug-related side effects were also reported. These included somnolence, headache, fatigue, dizziness, and lethargy. Somnolence had a significantly higher incidence with alprazolam compared to both placebo (*p* < 0.0001) and SB-649868 (*p* = 0.008), whereas fatigue was reported more frequently in the SB-649868 and alprazolam conditions, but was not significantly different between any of the groups at *p* = 0.05 (somnolence: placebo 4 %, SB-649868 17 %, alprazolam 54 %; fatigue: placebo 0 %, SB-649868 7 %, alprazolam 17 %). In contrast, headache and lethargy were reported more frequently in the placebo and SB-649868 conditions (headache: placebo 19 %, SB-649868 20 %, alprazolam 4 %; lethargy: placebo 4 %, SB-649868 3 %, alprazolam 0 %), and dizziness only with alprazolam (alprazolam 8 %); however, these effects were not significantly different between the conditions at *p* = 0.05.

### Neuroendocrine markers

**Fig. 2:**
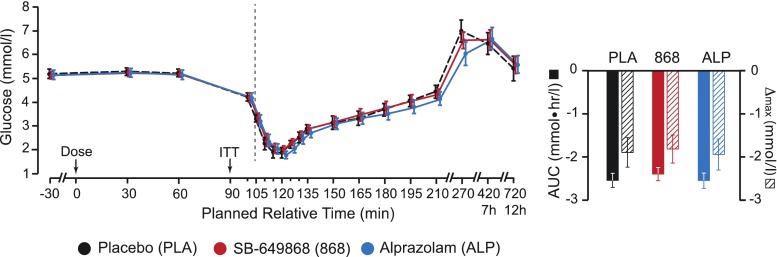
Plasma glucose concentration timecourses. *Left panel* shows group average glucose concentrations, across the three conditions: placebo, SB-649868, and alprazolam. *Right panel* shows area under curve (AUC) and maximum change from baseline (∆max) values (taken from the pre-ITT baseline, *t* = 90 min, until the end of data collection). *Error bars* represent 95 % confidence intervals

Mean pre-dose baseline levels (*t* = 0 min) of all neuroendocrine markers were not significantly different between drug conditions. In addition, the glucose response to ITT was similar regardless of the drug administered (Fig. 2). Thus, we concluded that any effects of SB-649868 and alprazolam, compared to placebo, must be a result of either drug administration or ITT.

**Fig. 3:**
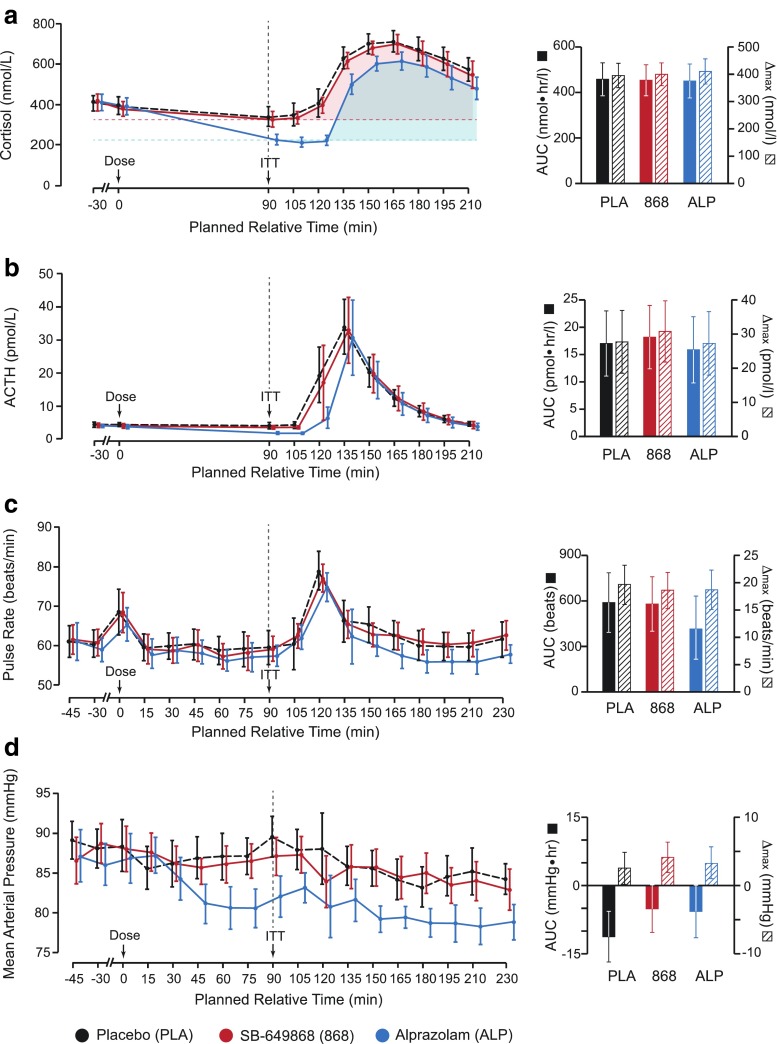
Summary of results from primary endpoints. Panels **a** and **b** show primary neuroendocrine markers; panels **c** and **d** show primary sympathetic nervous system markers. All *left panels* show group average timecourses under each of three conditions: placebo, SB-649868 and alprazolam. All *right panels* show area under curve (AUC) and maximum change from baseline (∆max) values, taken from the pre-ITT baseline, *t* = 90 min, until the end of data collection. *Error bars* represent 95 % confidence intervals. *Shaded regions* in **a** show how the AUC was calculated

For both primary neuroendocrine markers, plasma cortisol, and ACTH, we observed a marked increase in post-ITT plasma concentrations (relative to the pre-ITT baseline, *t* = 90 min), where subjects were pre-treated with placebo (AUC ± 95 % CI: ∆max ± 95 % CI; cortisol, 457.8 ± 72.5 nmol · hr/L: 395.8 ± 44.6 nmol/L; ACTH, 17.0 ± 6.0 pmol · hr/L: 27.7 ± 9.2 pmol/L). There was no significant difference to this AUC or ∆max response (see Methods section) when subjects were pre-treated with either SB-649868 or alprazolam (Fig. 3a, b). However, absolute pre-ITT baseline cortisol and ACTH levels were significantly reduced in response to alprazolam administration, compared to placebo (alprazolam-placebo, difference in adjusted mean score ± 95 % CI; cortisol, −101.1 ± 37.0 nmol/L, *p* < 0.0001; ACTH, −1.7 ± 0.4 pmol/L, *p* < 0.0001), and consequently, plasma concentrations of cortisol and ACTH at subsequent time points were significantly lower in the alprazolam condition than in the placebo condition (Fig. 3a, b).

**Fig. 4:**
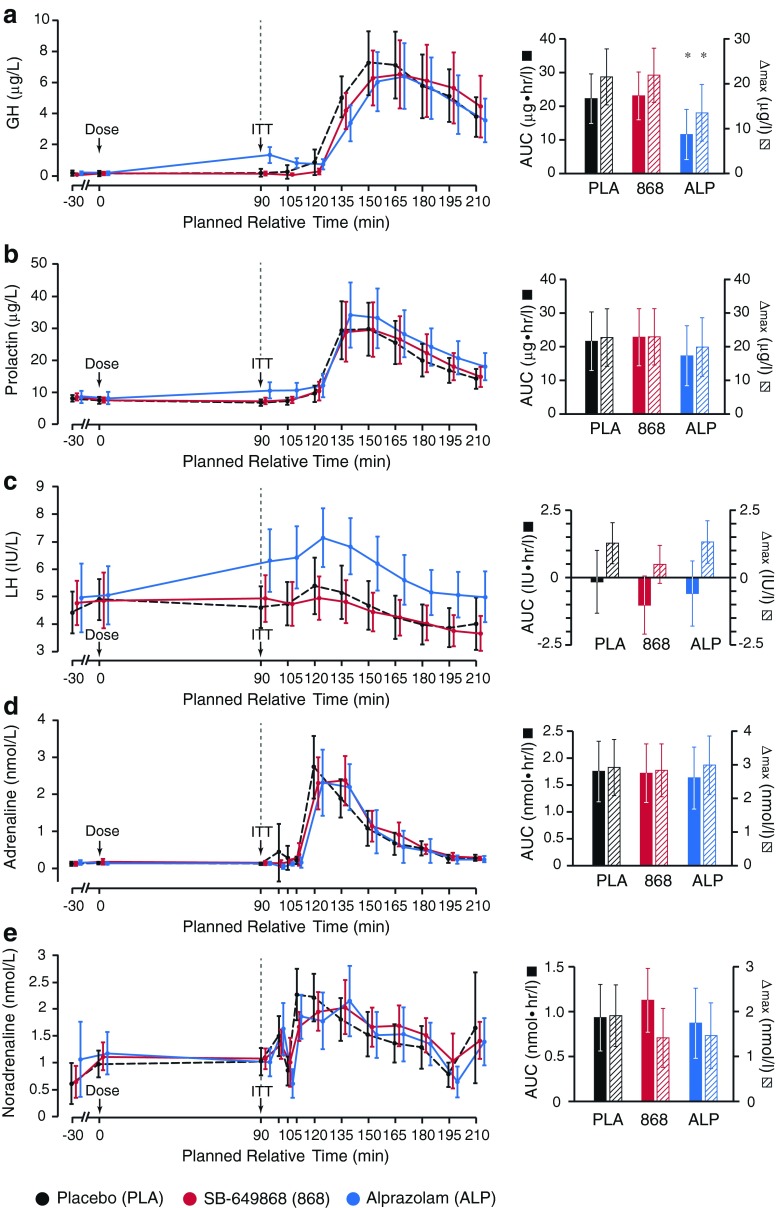
Summary of results from secondary endpoints. Panels **a**, **b**, and **c** show secondary neuroendocrine markers; panels **d** and **e** show secondary sympathetic nervous system markers. All *left panels* show group average timecourses under each of three conditions: placebo, SB-649868 and alprazolam. All *right panels* show area under curve (AUC) and maximum change from baseline plasma concentration (∆max) values, calculated from the pre-ITT baseline, *t* = 90 min, until the end of data collection. *Starred columns* represent data showing a significant difference from placebo; *error bars* represent 95 % confidence intervals

Of the secondary neuroendocrine markers (plasma GH, prolactin, and LH), a marked increase in plasma concentration was observed in response to ITT for both GH and prolactin, but not LH (AUC ± 95 % CI: ∆max ± 95 % CI; GH, 22.3 ± 7.4 μg · hr/L: 21.5 ± 6.2 μg/L; prolactin, 21.6 ± 8.7 μg · hr/L: 22.7 ± 8.6 μg/L; LH, −0.2 ± 1.2 IU · hr/L: 1.3 ± 0.8 IU/L). In addition, the pre-ITT baseline levels of GH and prolactin were significantly increased by alprazolam, but not SB-649868 (alprazolam-placebo, difference in adjusted mean score ± 95 % CI; GH, 1.1 ± 0.4 μg/L, *p* < 0.0001; prolactin, 3.3 ± 1.5 μg/L, *p* < 0.0001; Fig. 4a, b, c). Furthermore, pre-treatment with alprazolam significantly reduced the GH response to ITT, but this was not observed for SB-649868 (alprazolam-placebo, ∆AUC ± 95 % CI, −10.7 ± 6.2 μg · hr/L, *p* = 0.001; ∆max ± 95 % CI, −8.0 ± 5.2 μg/L, *p* = 0.003). Pre-treatment with alprazolam or SB-649868 had no significant impact on prolactin or LH responses to ITT (Fig. 4a, b, c).

### Sympathetic nervous system markers

As with the neuroendocrine markers, the pre-dose baseline levels (*t* = 0 min) of primary and secondary SNS markers were not significantly different between drug conditions.

Of the two primary SNS markers, pulse rate and MAP, a marked increase in pulse rate was observed in response to ITT (and also in response to drug administration at *t* = 0 min), reaching a maximum 30 min after insulin administration, in the placebo condition (AUC ± 95 % CI: ∆max ± 95 % CI, 589.3 ± 196.2 beats: 19.6 ± 3.6 beats/min; Fig. 3c). There were no synchronous changes in MAP, however, administration of alprazolam progressively reduced the mean baseline values of MAP, meaning that the pre-ITT baseline was significantly lower in the alprazolam condition than in the placebo condition (alprazolam-placebo, difference in adjusted mean score ± 95 % CI, −6.4 ± 2.8 mmHg, *p* < 0.0001; Fig. 3d). In analyzing the effects of SB-649868 and alprazolam on ITT-induced changes in pulse rate and MAP, we observed no significant difference from placebo, as measured by the AUC and ∆max .

**Fig. 5:**
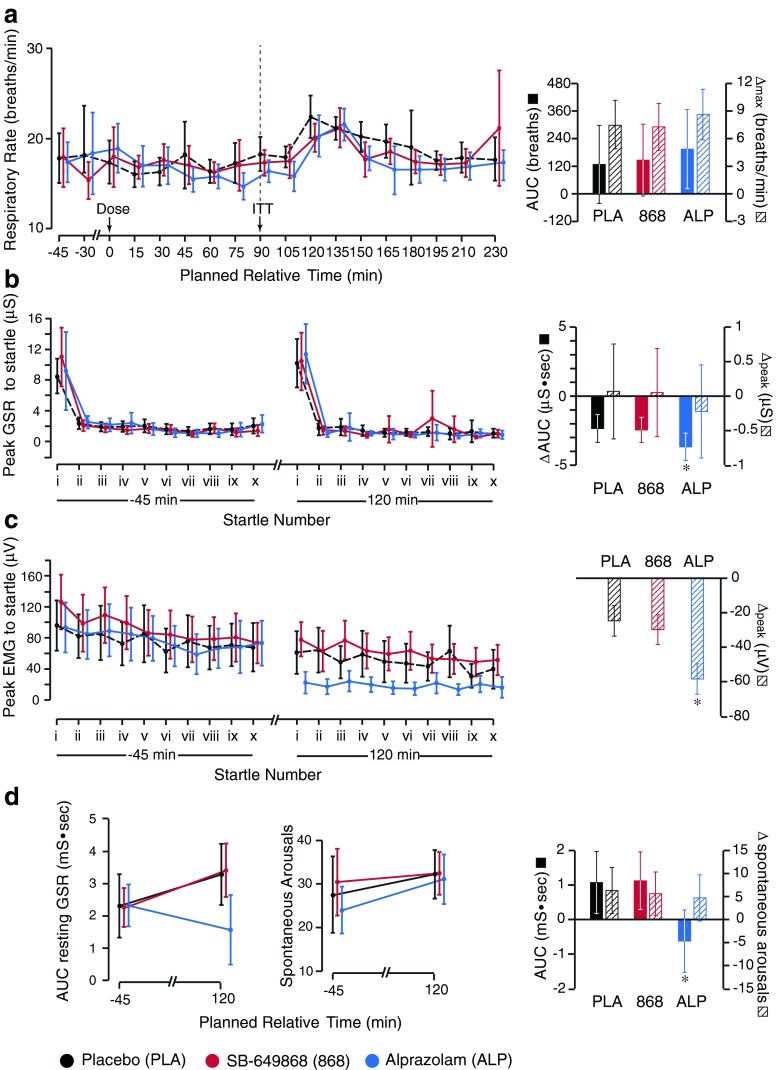
Summary of results from additional sympathetic nervous system markers. **a** *Left panel* shows group mean respiratory rate across the three conditions (placebo, SB-649868 and alprazolam), for the duration of the testing period. *Right panel* shows the area under curve (AUC) and maximum change from baseline (∆max), taken from the pre-ITT baseline, *t* = 90 min, until the end of data collection. **b** and **c** *Left panels* show the peak galvanic skin response (*GSR*) and electromyography (*EMG*) response, respectively, to acoustic startle, 45 min before dosing and 30 min after ITT. In each case, subjects were startled 10 times at pseudo-random intervals over 15 min. *Right panels* show the ΔAUC and Δpeak GSR (**b**), and Δpeak EMG (**c**) between the two time points (where values at each time point were averaged across the 10 trials). **d** *Left panel* shows results from the continuous 4 min resting GSR, 45 min before dosing and 30 min after ITT, represented as an area under the GSR vs. time curve over 4 min, for the three conditions. The *middle panel* shows the number of spontaneous arousals that occurred over that time period. **d** *Right panel* shows the ∆AUC and ∆max between the two time points. *Starred columns* highlight data that is significantly different from placebo; *error bars* represent 95 % confidence intervals

A number of secondary SNS markers were investigated: plasma adrenaline and NA, respiratory rate, resting GSR, and GSR and EMG responses to acoustic startle. ITT caused a marked increase in plasma concentration of adrenaline and NA, after pre-treatment with placebo, though this was more marked for adrenaline (AUC ± 95 % CI: ∆max ± 95 % CI; adrenaline, 1.7 ± 0.6 nmol · hr/L: 2.9 ± 0.8 nmol/L; NA, 77.3 ± 27.1 nmol · hr/L: 125.8 ± 41.7 nmol/L). Pre-treatment with alprazolam or SB-649868 resulted in no significant change from the placebo responses to ITT, nor did they significantly change pre-ITT baseline values of adrenaline and NA (Fig. 4d, e). Similarly, there was no significant difference between pre-treatment with SB-649868 or alprazolam, and placebo, with regards to mean respiratory rate (Fig. 5a). In addition, the mean respiratory rate did not significantly change in response to ITT, and the pre-ITT baseline was not significantly different between the three conditions.

In contrast, we observed a reduction from pre-dose values (*t* = −45 min) of AUC GSR (measured as the area under the curve for each startle response, averaged over 10 trials) and peak EMG (averaged over 10 trials) in response to startle, after ITT, in the placebo condition (*t* = 120 min; ∆AUC GSR ± 95 % CI, −2.3 ± 1.0 μS · sec; ∆*peak* EMG ± 95 % CI, −24.8 ± 8.9 μV; Fig. 5b, c). The post-ITT reduction in AUC GSR and peak EMG in response to startle was significantly larger than placebo when subjects were pre-treated with alprazolam, but not when subjects were pre-treated with SB-649868 (alprazolam-placebo; ∆AUC GSR ± 95 % CI, −1.3 ± 1.3 μS · sec, *p* = 0.04; ∆*peak* EMG ± 95 % CI, −33.7 ± 6.1 μV, *p* < 0.0001; Fig. 5b, c).

In the continuous 4-min resting GSR monitoring, while there was no significant difference in the number of spontaneous arousals before (*t* = −45 min) and after (*t* = 120 min) ITT, in any condition, and likewise no significant difference between conditions, pre-treatment with alprazolam resulted in a significant post-ITT reduction in AUC from the pre-dose baseline resting GSR AUC value, where the effect of pre-treatment with either placebo or SB-649868 was to marginally increase, or not affect, resting GSR after ITT (alprazolam-placebo, ∆AUC ± 95 % CI, 1.7 ± 1.3 mS · sec, *p* = 0.01; Fig. 5d).

### Behavioral markers

The following mood and behavioral markers were measured: Beck’s Anxiety Inventory (BAI); Appetite (Visual Analogue Scale, VAS); Hypoglycemic symptoms (VAS); Hunger, Craving, and Fullness Questionnaire (HCFQ); and the quantity and rate of food consumption upon completion of the ITT. Pre-treatment with alprazolam significantly decreased appetite post-ITT, compared to placebo, as measured by the appetite VAS, at all time points analyzed (alprazolam-placebo, difference in adjusted mean score ± 95 % CI; *t* = 135 min, −55.7 ± 31.1 mm, *p* = 0.0005; *t* = 180 min, −62.9 ± 31.1 mm, *p* < 0.0001; *t* = 210 min, −58.2 ± 31.1 mm, *p* = 0.0003). There was no significant difference between pre-treatment conditions for any other behavioral endpoints (Supplementary Fig. 1).

### Pharmacokinetic assays

The concentration of SB-649868 was assayed at four time points after dosing. A peak in mean plasma concentration was observed 30 min after ITT (*t* = 120 min; Supplementary Fig. 2a), which corresponded to peak values for most primary and secondary endpoints. When subjects were pre-treated with SB-649868, a corresponding decrease in plasma concentration of orexin A was observed, though this was not significantly different to placebo (Supplementary Fig. 2b; orexin A levels assayed by Pharmidex UK). No analogous decrease was observed in the alprazolam condition. In addition, plasma orexin A concentrations were not significantly changed in response to ITT in any condition.

## Discussion

In this study, we attempted to validate the insulin tolerance test (ITT) model for studying orexin antagonist activity in response to hypoglycemic stress in healthy adult humans. A summary of the effect sizes of pre-treatment with the positive control, alprazolam, and the study compound, SB-649868, on neuroendocrine and sympathetic nervous system responses to ITT can be found in Table 1.

**Table 1:** Summary of effect sizes for primary and secondary, neuroendocrine and sympathetic nervous system markers

	Neuroendocrine					Sympathetic nervous system				
	Endpoint	SB-649868		Alprazolam		Endpoint	SB-649868		Alprazolam	
		ΔAUC	Δmax	ΔAUC	Δmax		ΔAUC	Δmax	ΔAUC	Δmax
Primary	Cortisol	−0.03	0.05	−0.05	0.15	Pulse Rate	−0.03	−0.16	−0.41	−0.15
	ACTH	0.15	0.28	−0.15	−0.04	MAP	0.45	0.28	0.40	0.12
Secondary	GH	0.08	0.04	**−1.02**	**−0.93**	Adrenaline	−0.05	−0.07	−0.20	0.06
	Prolactin	0.10	0.02	−0.37	−0.27	NA	0.25	−0.28	−0.08	−0.25
	LH	−0.29	−0.41	−0.15	0.02	Resp. rate	0.05	−0.03	0.17	0.24
	Glucose	0.11		−0.07		Resting GSR	0.02	−0.07	**−0.78**	−0.17
						Startle GSR	−0.10	0.00	**−0.19**	−0.08
						Startle EMG		−0.16		**−1.07**

Bold figures represent endpoints showing a significant difference from placebo

In the placebo condition, ITT markedly increased plasma concentrations of most neuroendocrine hormones (cortisol, ACTH, GH, and prolactin), and most SNS markers (pulse rate, plasma noradrenaline and adrenaline, and EMG / GSR responses to acoustic startle), suggesting that the ITT model may be of great utility for analyzing the pharmacological efficacy of compounds acting directly or indirectly on these systems.

Pre-treatment with alprazolam had a number of effects on primary and secondary endpoints. First, alprazolam appeared to affect almost all neuroendocrine markers from the time of dosing, such that at *t* = 90 min (the start of ITT), mean levels of cortisol, ACTH, and MAP, were significantly lower than in the placebo condition, and conversely, plasma concentrations of GH, prolactin, and LH were significantly higher. These findings are consistent with a previous experimental study on the effects of alprazolam on neuroendocrine responses to ITT (Giordano et al. 2003). The change in baseline after dosing contributed a significant confound to subsequent analyses, which all took pre-ITT values (*t* = 90 min) as a reference point. For endpoints where pre-ITT measurements were not taken, and pre-dose values were used as baselines (resting GSR, and EMG/GSR response to startle), this confound was not apparent, and alprazolam pre-treatment was found to significantly reduce post-ITT responses in all of these markers, compared to placebo. The fact that alprazolam was able to effect changes in baseline levels of neuroendocrine and SNS markers suggests that the ITT model, which stimulated increases in these markers, may be useful for investigating the efficacy of alprazolam, or more generally, the role of GABA receptors in the pituitary and adrenal medulla; however, the experimental design may need to be modified to account for the effects observed immediately after compound administration. Furthermore, the results reported in our study following 0.02 mg/kg alprazolam administration were broadly, but not entirely, consistent with those reported in Giordano et al. (2003), which found that alprazolam pre-treatment significantly reduced ITT-induced increases in ACTH and plasma adrenaline, in addition to GH. We hypothesize that this most likely reflects differences in the cohorts analyzed.

The orexin-hypocretin system, by contrast, appeared to have no significant impact on any endpoints measured; our findings can be summarized into two main results. First, in the placebo condition, orexin A levels were approximately constant throughout the experimental procedure, with no significant change in plasma orexin A concentration in response to ITT (Supplementary Fig. 2B, black line). This suggests that either peripheral orexin A is not a good marker of central nervous system (CNS) orexinergic neuron activity, our assay method for orexin A was not sensitive enough to detect small changes in plasma orexin A, or that the ITT model was unable to stimulate high endogenous agonist release. Assaying CNS signaling non-invasively, in general, presents a challenge; in this study, subtle changes in CNS orexinergic neuron activity might not have been well detected by peripheral markers, such as plasma orexin A, and of course the measurements may have been confounded by the activity of peripheral orexinergic neurons. In addition, while there is substantial evidence to suggest that hypoglycemia can robustly activate CNS orexin signaling in vitro and in rodents, it is possible that this does not translate well to humans, and that the method for hypoglycemia-induced increase in HPA axis activation is due in part to a different mechanism. Secondly, pre-treatment with SB-649868 had no significant impact on any neuroendocrine, SNS, or behavioral responses to ITT, nor were any significant changes to pre-dose baseline values observed after dosing. If CNS orexin levels were not adequately stimulated by hypoglycemic challenge, this result would not be entirely surprising. In addition, although SB-649868 is known to be a potent insurmountable antagonist at both OxR1 and OxR2 receptors (Faedo et al. 2012), previous in vivo studies reporting increased plasma corticosterone (Hagan et al. 1999; Kuru et al. 2000) or plasma ACTH in response to orexin administration in rodents, administered orexin centrally. By extension, it is therefore possible that the local concentration of SB-649868 at CNS OxR1 and OxR2 receptors was insufficient to have any pharmacological effect even if central orexin signaling had been adequately stimulated by hypoglycemia; in other words, the compound had poor antagonist efficacy at the bioavailable dose. The lack of antagonist efficacy may also have been exacerbated by a lack of antagonist affinity at human orexin receptors, or by any potential antagonistic effects of CNS versus peripheral orexin neurons. We hypothesize that any combination of these factors could have contributed to a lack of significant results produced by the orexin-hypocretin system in response to hypoglycemic challenge in this study. However, we also note that this study was motivated specifically by the intention to investigate the pharmacodynamic efficacy of SB-649868 at doses lower than the dose range previously associated with hypnotic efficacy, i.e., < 20 mg. In future studies, it would be interesting to explore the utility of the ITT model as a basis for evaluating orexinergic antagonism over a wider dose range including doses known to have efficacy on other markers, such as measures of insomnia. It remains tenable that orexin antagonists will have the theoretically anticipated effect on neuroendocrine responses to hypoglycemia at higher doses; but it was beyond the scope of this study to explore a range of higher doses of this molecule.

Finally, there is some evidence that OxR1 and OxR2 receptors may have complementary roles relating to their tissue expression, and this may underlie their different roles in gating REM and non-REM sleep (Mieda et al. 2011). Therefore, the development of selective receptor antagonists may be beneficial for future experimental medicine studies.

## Conclusion

In conclusion, we show that alprazolam reduces some neuroendocrine and sympathetic nervous system responses to insulin-induced hypoglycemia and, furthermore, that this model may be valuable for further assessing the role of GABAergic pathways in the pituitary and adrenal medulla. However, hypoglycemia did not appear to stimulate orexinergic neuron activity (as measured by peripheral orexin A levels), nor did low dose SB-649868 have a significant effect on any endpoint, and therefore the ITT model could not be validated with regard to investigating pharmacotherapeutic effects on orexinergic pathways. These findings may be particularly relevant in light of recent FDA decisions to only grant approval for low dose studies of a similar dual OxR1 and OxR2 antagonist, Suvorexant.

## Electronic supplementary material

Below is the link to the electronic supplementary material.

Supplementary Fig. 1 (DOC 61 kb)

Supplementary Fig. 2 (DOCX 40 kb)
