# Supplementary material for: Neuroendocrine and sympathetic responses to an orexin receptor antagonist, SB-649868, and Alprazolam following insulin-induced hypoglycemia in humans
Source: Psychopharmacology (Berl). 2014 Apr 26;231(19):3817–28. doi: 10.1007/s00213-014-3520-7 (PMC4159598; doi:10.1007/s00213-014-3520-7)
Supplement: Supplementary file 1 — (DOC 61 kb) [file 213_2014_3520_MOESM1_ESM.doc]

**Supplementary Information**

**Supplementary Fig. 1** *Summary of results from behavioral markers.* For (A) and (C), data was collected at four time points: *t*=105, 135, 165, and 210 min. For (B) and (D), data was collected at three time points: *t*=135, 180, 210 min. Starred columns with solid fill represent data showing a significant difference from placebo; error bars represent 95% confidence intervals.
