# Supplementary material for: Neuroendocrine and sympathetic responses to an orexin receptor antagonist, SB-649868, and Alprazolam following insulin-induced hypoglycemia in humans
Source: Psychopharmacology (Berl). 2014 Apr 26;231(19):3817–28. doi: 10.1007/s00213-014-3520-7 (PMC4159598; doi:10.1007/s00213-014-3520-7)
Supplement: Supplementary file 2 — (DOCX 40 kb) [file 213_2014_3520_MOESM2_ESM.docx]

**Supplementary Fig. 2** *Summary of results from pharmacokinetic assays.* (A) shows the average plasma concentration of orexin receptor antagonist, SB-649868, at four time points after compound administration. (B) shows mean plasma orexin A concentrations for the study period, across all three conditions (placebo, SB-649869 and Alprazolam). Error bars represent 95% confidence intervals.
